# The Probabilities of Trees and Cladograms under Ford's α-Model

**DOI:** 10.1155/2018/1916094

**Published:** 2018-04-18

**Authors:** Tomás M. Coronado, Arnau Mir, Francesc Rosselló

**Affiliations:** Balearic Islands Health Research Institute (IdISBa) and Department of Mathematics and Computer Science, University of the Balearic Islands, 07122 Palma, Spain

## Abstract

Ford's *α*-model is one of the most popular random parametric models of bifurcating phylogenetic tree growth, having as specific instances both the uniform and the Yule models. Its general properties have been used to study the behavior of phylogenetic tree shape indices under the probability distribution it defines. But the explicit formulas provided by Ford for the probabilities of unlabeled trees and phylogenetic trees fail in some cases. In this paper we give correct explicit formulas for these probabilities.

## 1. Introduction

The study of random growth models of rooted phylogenetic trees and the statistical properties of the shapes of the phylogenetic trees they produce was initiated almost one century ago by Yule [1] and it has gained momentum in the last 20 years: see, for instance, [2–8]. The final goal of this line of research is to understand the relationship between the forces that drive evolution and the topological properties of “real-life” phylogenetic trees [3, 9]; see also [10, Chapter 33]. One of the most popular such models is Ford's *α*-model for rooted bifurcating phylogenetic trees or* cladograms* [4]. It consists of a parametric model that generalizes both the uniform model (where new leaves are added equiprobably to any arc, giving rise to the uniform probability distribution on the sets of cladograms with a fixed set of taxa) and Yule's model (where new leaves are added equiprobably only to* pendant* arcs, i.e., to arcs ending in leaves) by allocating a possibly different probability (that depends on a parameter *α* and hence its name, “*α*-model”) to the addition of the new leaves to pendant arcs or to internal arcs.

When models like Ford's model are used to contrast topological properties of phylogenetic trees contained in databases like TreeBase (https://treebase.org), only their general properties (moments, asymptotic behavior) are employed. But, in the course of a research where we have needed to compute the probabilities of several specific cladograms under this model [11], we have noticed that the explicit formulas that Ford gives in [4, §3.5] for the probabilities of cladograms and of* tree shapes* (unlabeled rooted bifurcating trees) are wrong, failing for some trees with *n*⩾8 leaves; see Propositions 29 and 32 in [4], with the definition of $\hat{q}$ given in page 30 therein, for Ford's formulas.

So, to help the future user of Ford's model, in this paper we give the correct explicit formulas for these probabilities. This paper is accompanied by the GitHub page https://github.com/biocom-uib/prob-alpha where the interested reader can find a SageMath [12] module to compute these probabilities and their explicit values on the sets *𝒯*_*n*_ of cladograms with *n* leaves labeled 1,…, *n*, for every *n* from 2 to 8.

## 2. Preliminaries

### 2.1. Definitions, Notations, and Conventions

Throughout this paper, by a* tree T*, we mean a rooted bifurcating tree. As it is customary, we understand *T* as a directed graph, with its arcs pointing away from the root, which we shall denote by *r*_*T*_. Then, all nodes in *T* have out-degree either 0 (its* leaves*, which form the set *L*(*T*)) or 2 (its* internal nodes*, which form the set *V*_int_(*T*)). The* children* of an internal node *v* are those nodes *u* such that (*v*, *u*) is an arc in *T*, and they form the set child(*v*). A node *x* is a* descendant* of a node *v* when there exists a directed path from *v* to *x* in *T*. For every node *v*, the* subtree T*_*v*_ *of T rooted at v* is the subgraph of *T* induced on the set of descendants of *v*.

A tree *T* is* ordered* when it is endowed with an* ordering *≺_*v*_ on every set child(*v*). A* cladogram* (resp., an* ordered cladogram*) on a set of taxa Σ is a tree (resp., an ordered tree) with its leaves bijectively labeled in Σ. Whenever we want to stress the fact that a tree is not a cladogram, that is, it is an unlabeled tree, we shall use the term* tree shape*.

It is important to point out that although ordered trees have no practical interest from the phylogenetic point of view, because the orderings on the sets of children of internal nodes do not carry any phylogenetic information, they are useful from the mathematical point of view, because the existence of the orderings allows one to easily prove certain extra properties that can later be translated to the unordered setting (cf.  Proposition 1).

An* isomorphism* of ordered trees is an isomorphism of rooted trees that moreover preserves these orderings. An* isomorphism* of cladograms (resp., of ordered cladograms) is an isomorphism of trees (resp., of ordered trees) that preserves the leaves' labels. We shall always identify a tree shape, an ordered tree shape, a cladogram, or an ordered cladogram, with its isomorphism class, and in particular we shall make henceforth the abuse of language of saying that two of these objects, *T*, *T*′,* are the same*, in symbols *T* = *T*′, when they are (only) isomorphic. We shall denote by *𝒯*_*n*_^*∗*^ and *𝒪𝒯*_*n*_^*∗*^, respectively, the sets of tree shapes and of ordered tree shapes with *n* leaves. Given any finite set of taxa Σ, we shall denote by *𝒯*_Σ_ and *𝒪𝒯*_Σ_, respectively, the sets of cladograms and of ordered cladograms on Σ. When the specific set Σ is unrelevant and only its cardinal matters, we shall write *𝒯*_*n*_ and *𝒪𝒯*_*n*_ (with *n* = |Σ|) instead of *𝒯*_Σ_ and *𝒪𝒯*_Σ_, and then we shall understand that Σ is [*n*] = {1,2,…, *n*}.

**Figure 1:**
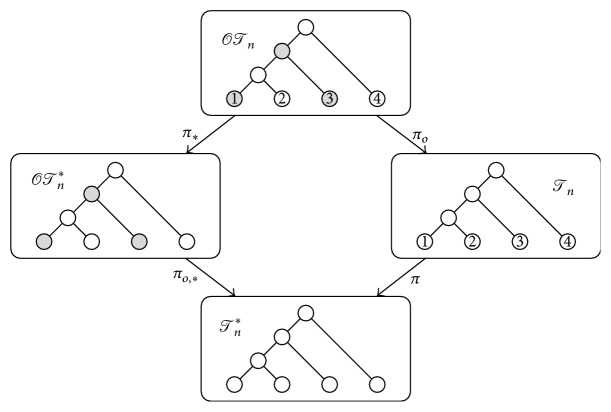
An example of images under the forgetful mappings between (ordered and unordered) cladograms and tree shapes. In the ordered objects, the ordering is represented by the nodes' colors: gray ≺ white.

There exist natural isomorphism-preserving forgetful mappings
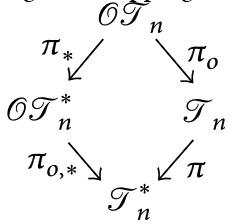


 that “forget” the orderings or the labels of the trees. In particular, we shall call the image of a cladogram under *π* its* shape*.  Figure 1 depicts an example of images under these forgetful mappings.

**Figure 2:**
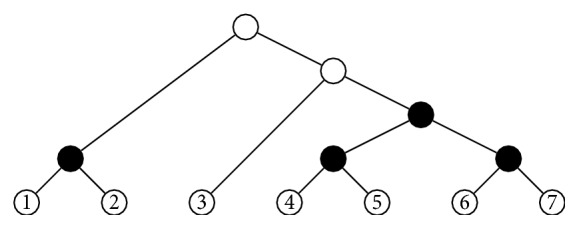
A cladogram in *𝒯*_7_. The black nodes are its symmetric branch points.

Let us introduce some more notations. For every node *v* in a tree *T*, *κ*_*T*_(*v*) is its number of descendant leaves. For every internal node *v* in an ordered tree *T*, with children *v*_1_≺_*v*_ *v*_2_, its* numerical split* is the ordered pair NS_*T*_(*v*) = (*κ*_*T*_(*v*_1_), *κ*_*T*_(*v*_2_)). If, instead, *T* is unordered and if child(*v*) = {*v*_1_, *v*_2_} with *κ*_*T*_(*v*_1_) ⩽ *κ*_*T*_(*v*_2_), then NS_*T*_(*v*) = (*κ*_*T*_(*v*_1_), *κ*_*T*_(*v*_2_)). In both cases, the* multiset of numerical splits* of *T* is NS(*T*) = {NS_*T*_(*v*)∣*v* ∈ *V*_int_(*T*)}. For instance, if *T* is the cladogram depicted in Figure 2, then

(1) $$\begin{array}{c} NS\left(\;T\;\right)=\left\{\;\left(\;1,1\;\right),\left(\;1,1\;\right),\left(\;1,1\;\right),\left(\;2,2\;\right),\left(\;1,4\;\right),\left(\;2,5\;\right)\;\right\}. \end{array}$$

A* symmetric branch point* in a tree *T* is an internal node *v* such that if *v*_1_ and *v*_2_ are its children, then the subtrees *T*_*v*_1__ and *T*_*v*_2__ of *T* rooted at them have the same shape. For instance, the symmetric branch points in the cladogram depicted in Figure 2 are those filled in black.

**Figure 3:**
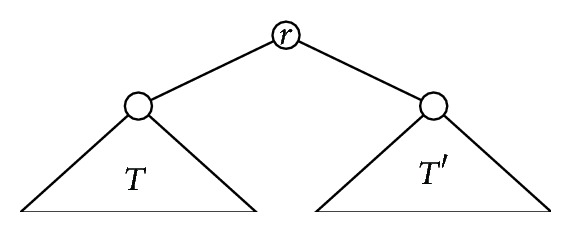
The root join *T*⋆*T*′.

Given two cladograms *T* and *T*′ on Σ and Σ′, respectively, with Σ∩Σ′ = *∅*, their* root join* is the cladogram *T*⋆*T*′ on Σ ∪ Σ′ obtained by connecting the roots of *T* and *T*′ to a (new) common root *r*; see Figure 3. If *T*, *T*′ are ordered cladograms, *T*⋆*T*′ is ordered by inheriting the orderings on *T* and *T*′ and ordering the children of the new root *r* as *r*_*T*_≺_*r*_ *r*_*T*′_. If *T* and *T*′ are tree shapes, a similar construction yields a tree shape *T*⋆*T*′; if they are moreover ordered, then *T*⋆*T*′ becomes an ordered tree shape as explained above.

### 2.2. The *α*-Model

Ford's *α*-model [4] defines, for every *n*⩾1, a family of probability density functions *P*_*α*,*n*_^(*∗*)^ on *𝒯*_*n*_^*∗*^ that depends on one parameter *α* ∈ [0,1], and then it translates this family into three other families of probability density functions *P*_*α*,*n*_ on *𝒯*_*n*_, *P*_*α*,*n*_^(*o*,*∗*)^ on *𝒪𝒯*_*n*_^*∗*^, and *P*_*α*,*n*_^(*o*)^ on *𝒪𝒯*_*n*_, by imposing that the probability of a tree shape is equally distributed among its preimages under *π*, *π*_*o*,*∗*_, and *π*∘*π*_*o*_ = *π*_*o*,*∗*_∘*π*_*∗*_, respectively.

It is well known [13] that every *T* ∈ *𝒯*_*n*_ can be obtained in a unique way by adding recurrently to a single node labeled 1 new leaves labeled 2,…, *n* to arcs (i.e., splitting an arc (*u*, *v*) into two arcs (*u*, *w*) and (*w*, *v*) and then adding a new arc from the inserted node *w* to a new leaf) or to a new root (i.e., adding a new root *w* and new arcs from *w* to the old root and to a new leaf). The value of *P*_*α*,*n*_^(*∗*)^(*T* ^*∗*^) for *T* ^*∗*^ ∈ *𝒯*_*n*_^*∗*^ is determined through all possible ways of constructing cladograms with shape *T* ^*∗*^ in this way. More specifically,

(1) if *T*_1_ and *T*_2_ denote, respectively, the only cladograms in *𝒯*_1_ and *𝒯*_2_, let *P*_*α*,1_′(*T*_1_) = *P*_*α*,2_′(*T*_2_) = 1;
(2) for every *m* = 3,…, *n*, let *T*_*m*_ ∈ *𝒯*_*m*_ be obtained by adding a new leaf labeled *m* to *T*_*m*−1_. Then
  (2) $$\begin{array}{c} P_{\alpha ,m}^{\prime }\left(\;T_{m}\;\right)=\left\{\begin{array}{cc} \frac{\alpha }{m-1-\alpha }\cdot P_{\alpha ,m-1}^{\prime }\left(T_{m-1}\right) & \text{if the new leaf is added to an internal arc or to a new root}\\ \frac{1-\alpha }{m-1-\alpha }\cdot P_{\alpha ,m-1}^{\prime }\left(T_{m-1}\right) & \text{if the new leaf is added to a pendant arc;} \end{array}\right. \end{array}$$
(3) When the desired number *n* of leaves is reached, the probability of every tree shape *T*_*n*_^*∗*^ ∈ *𝒯*_*n*_^*∗*^ is defined as
  (3) $$\begin{array}{c} P_{\alpha ,n}^{\left(*\right)}\left(\;T_{n}^{*}\;\right)=\underset{\pi \left(T_{n}\right)=T_{n}^{*}}{\sum }P_{\alpha ,n}^{\prime }\left(\;T_{n}\;\right). \end{array}$$

**Figure 4:**
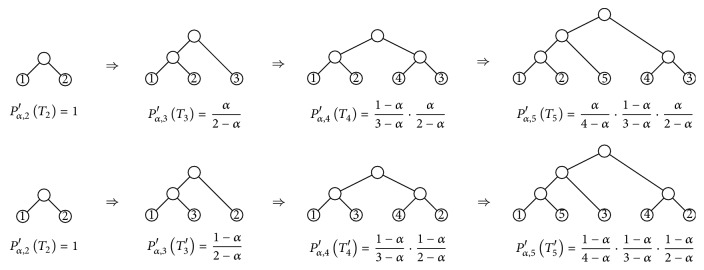
Two examples of computations of the probability *P*_*α*,*n*_′ of a cladogram through its construction in Step (2) of the definition of the *α*-model.

For instance, Figure 4 shows the construction of two cladograms in *𝒯*_5_ with the same shape and how their probability *P*_*α*,5_′ is built using the recursion in Step (2). If we generate all cladograms in *𝒯*_5_ with this shape, we compute their probabilities *P*_*α*,5_′, and then we add up all these probabilities, we obtain the probability *P*_*α*,5_^(*∗*)^ of this shape, which turns out to be 2(1 − *α*)/(4 − *α*); cf. [4, Figure 23].

Once *P*_*α*,*n*_^(*∗*)^ is defined on *𝒯*_*n*_^*∗*^, it is transported to *𝒯*_*n*_, *𝒪𝒯*_*n*_^*∗*^, and *𝒪𝒯*_*n*_ by defining the probability of an object in one of these sets as the probability of its image in *𝒯*_*n*_^*∗*^ divided by the number of preimages of this image:

(i) For every *T* ∈ *𝒯*_*n*_, if *π*(*T*) = *T* ^*∗*^ ∈ *𝒯*_*n*_^*∗*^ and it has *k* symmetric branch points, then
  (4) $$\begin{array}{c} P_{\alpha ,n}\left(\;T\;\right)=\frac{2^{k}}{n!}\cdot P_{\alpha ,n}^{\left(*\right)}\left(\;T^{*}\;\right), \end{array}$$
because |*π* ^−1^(*T* ^*∗*^)| = *n*!/2^*k*^ (see, e.g., [4, Lemma 31]).
(ii) For every *T*_*o*_ ∈ *𝒪𝒯*_*n*_, if *π*_*o*_(*T*_*o*_) = *T* ∈ *𝒯*_*n*_, then
  (5) $$\begin{array}{c} P_{\alpha ,n}^{\left(o\right)}\left(\;T_{o}\;\right)=\frac{1}{2^{n-1}}\cdot P_{\alpha ,n}\left(\;T\;\right), \end{array}$$
because |*π*_*o*_^−1^(*T*)| = 2^*n*−1^ (*T* has 2^*n*−1^ different preimages under *π*_*o*_, obtained by taking all possible different combinations of orderings on the *n* − 1 sets child(*v*), *v* ∈ *V*_int_(*T* ^*∗*^)).
(iii) For every *T*_*o*_^*∗*^ ∈ *𝒪𝒯*_*n*_^*∗*^, if *π*_*o*,*∗*_(*T*_*o*_^*∗*^) = *T* ^*∗*^ ∈ *𝒯*_*n*_^*∗*^ and it has *k* symmetric branch points, then
  (6) $$\begin{array}{c} P_{\alpha ,n}^{\left(o,*\right)}\left(\;T_{o}^{*}\;\right)=\frac{1}{2^{n-k-1}}\cdot P_{\alpha ,n}^{\left(*\right)}\left(\;T^{*}\;\right), \end{array}$$
because |*π*_*o*,*∗*_^−1^(*T* ^*∗*^)| = 2^*n*−1−*k*^ (from the 2^*n*−1^ possible preimages of *T* ^*∗*^ under *π*_*o*,*∗*_, defined by all possible different combinations of orderings on the *n* − 1 sets child(*v*), *v* ∈ *V*_int_(*T* ^*∗*^), those differing only on the orderings on the children of the *k* symmetric branch points are actually the same ordered tree shape).

The family (*P*_*α*,*n*_^(*o*,*∗*)^)_*n*_ of probabilities of ordered tree shapes satisfies the useful Markov branching recurrence (in the sense of [2, §4]) given by the following proposition. In it and in the sequel, let, for every *a*, *b* ∈ *ℤ* ^+^,

(7) $$\begin{array}{c} q_{\alpha }\left(\;a,b\;\right)=\frac{\Gamma_{\alpha }\left(a\right)\Gamma_{\alpha }\left(b\right)}{\Gamma_{\alpha }\left(a+b\right)}\cdot \varphi_{\alpha }\left(\;a,b\;\right), \end{array}$$

where

(8) $$\begin{array}{c} \varphi_{\alpha }\left(\;a,b\;\right)=\frac{\alpha }{2}\left(\begin{array}{c} a+b\\ a \end{array}\right)+\left(\;1-2\alpha \;\right)\left(\begin{array}{c} a+b-2\\ a-1 \end{array}\right) \end{array}$$

and Γ_*α*_ : *ℤ* ^+^ → *ℝ* is the mapping defined by Γ_*α*_(1) = 1 and, for every *n*⩾2, Γ_*α*_(*n*) = (*n* − 1 − *α*) · Γ_*α*_(*n* − 1).

**Proposition 1 .**

For every 0 < *m* < *n* and for every *T*_*m*_^*∗*^ ∈ *𝒪𝒯*_*m*_^*∗*^ and *T*_*n*−*m*_^*∗*^ ∈ *𝒪𝒯*_*n*−*m*_^*∗*^,

(9) Pα,no,∗Tm∗⋆Tn−m∗=qαm,n−mPα,mo,∗Tm∗Pα,n−mo,∗Tn−m∗.

This recurrence, together with the fact that *P*_*α*,1_^(*o*,*∗*)^ of a single node is 1, implies that, for every *T*_*o*_^*∗*^ ∈ *𝒪𝒯*_*n*_^*∗*^,

(10) $$\begin{array}{c} P_{\alpha ,n}^{\left(o,*\right)}\left(\;T_{o}^{*}\;\right)=\underset{\left(a,b\right)\in NS\left(T_{o}^{*}\right)}{\prod }q_{\alpha }\left(\;a,b\;\right). \end{array}$$

For proofs of Proposition 1 and (10), see Lemma 27 and Proposition 28 in [4], respectively.

## 3. Main Results

Our first result is an explicit formula for *P*_*α*,*n*_(*T*), for every *n*⩾1 and *T* ∈ *𝒯*_*n*_.

**Proposition 2 .**

For every *T* ∈ *𝒯*_*n*_, its probability under the *α*-model is

(11) $$\begin{array}{c} P_{\alpha ,n}\left(\;T\;\right)=\frac{2^{n-1}}{n!\cdot \Gamma_{\alpha }\left(n\right)}\underset{\left(a,b\right)\in NS\left(T\right)}{\prod }\varphi_{\alpha }\left(\;a,b\;\right). \end{array}$$

**Proof**

Given *T* ∈ *𝒯*_*n*_, let *T*_*o*_ be any ordered cladogram such that *π*_*o*_(*T*_*o*_) = *T*, and let *T*_*o*_^*∗*^ = *π*_*∗*_(*T*_*o*_) ∈ *𝒪𝒯*_*n*_^*∗*^ and *T* ^*∗*^ = *π*(*T*) = *π*_*o*,*∗*_(*T*_*o*_^*∗*^). If *T* ^*∗*^ has *k* symmetric branch points, then, by (4), (6), and (10),

(12) Pα,nT=2kn!·Pα,n∗T∗=2kn!·2n−k−1·Pα,no,∗To∗=2n−1n!∏a,b∈NSTo∗qαa,b.

Now, on the one hand, it is easy to check that

(13) NST=min⁡a,b,max⁡a,b ∣ a,b∈NST0∗,

and therefore, since *q*_*α*_ is symmetric,

(14) $$\begin{array}{c} P_{\alpha ,n}\left(\;T\;\right)=\frac{2^{n-1}}{n!}\underset{\left(a,b\right)\in NS\left(T\right)}{\prod }q_{\alpha }\left(\;a,b\;\right). \end{array}$$

It remains to simplify this product. If, for every *v* ∈ *V*_int_(*T*), we denote its children by *v*_1_ and *v*_2_, then

(15) ∏a,b∈NSTqαa,b=∏v∈VintTΓακTv1ΓακTv2ΓακTvφαNSv.

For every *v* ∈ *V*_int_(*T*)∖{*r*_*T*_}, the term Γ_*α*_(*κ*_*T*_(*v*)) appears twice in this product: in the denominator of the factor corresponding to *v* itself and in the numerator of the factor corresponding to its parent. Therefore, all terms Γ_*α*_(*κ*_*T*_(*v*)) in this product vanish except Γ_*α*_(*κ*_*T*_(*r*_*T*_)) = Γ_*α*_(*n*) (that appears in the denominator of its factor) and every Γ_*α*_(*κ*_*T*_(*v*)) = Γ_*α*_(1) = 1 with *v*, a leaf. Thus,

(16) $$\begin{array}{c} P_{\alpha ,n}\left(\;T\;\right)=\frac{2^{n-1}}{n!}\cdot \frac{1}{\Gamma_{\alpha }\left(n\right)}\cdot \underset{v\in V_{int}\left(T\right)}{\prod }\varphi_{\alpha }\left(\;NS\left(\;v\;\right)\;\right) \end{array}$$

as we claimed.

**Remark 3 .**

Ford states (see [4, Proposition  32 and page 30]) that if *T* ∈ *𝒯*_*n*_, then

(17) $$\begin{array}{c} P_{\alpha ,n}\left(\;T\;\right)=\frac{2^{k}}{n!}\underset{\left(a,b\right)\in NS\left(T\right)}{\prod }{\hat{q}}_{\alpha }\left(\;a,b\;\right), \end{array}$$

where *k* is the number of symmetric branching points in *T* and

(18) $$\begin{array}{c} {\hat{q}}_{\alpha }\left(\;a,b\;\right)=\left\{\begin{array}{cc} 2q_{\alpha }\left(a,b\right) & \text{if }a\neq b\\ q_{\alpha }\left(a,b\right) & \text{if }a=b. \end{array}\right. \end{array}$$

If we simplify $\prod_{\left(a,b)\in NS(T\right)}{\hat{q}}_{\alpha }(a,b)$ as in the proof of Proposition 2, this formula for *P*_*α*,*n*_(*T*) becomes

(19) $$\begin{array}{c} P_{\alpha ,n}\left(\;T\;\right)=\frac{2^{k+m}}{n!\cdot \Gamma_{\alpha }\left(n\right)}\cdot \underset{\left(a,b\right)\in NS\left(T\right)}{\prod }\varphi_{\alpha }\left(\;a,b\;\right), \end{array}$$

where *m* is the number of internal nodes whose children have different numbers of descendant leaves. This formula does not agree with the one given in Proposition 2 above, because

**Figure 5:**
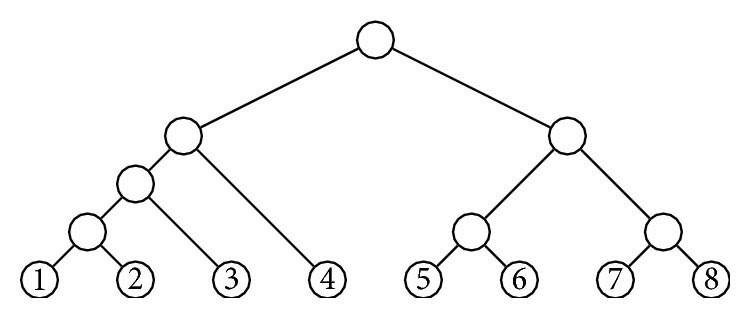
The cladogram $\tilde{T}\in {𝒯}_{8}$ used in Remark 3.

(20) k+m=n−1−v∈VintT ∣ childv=v1,v2,  κTv1=κTv2  but  πTv1≠πTv2

and, hence, it may happen that *k* + *m* < *n* − 1. The first example of a cladogram with this property (and the only one, up to relabeling, with at most 8 leaves) is the cladogram $\tilde{T}\in {𝒯}_{8}$ depicted in Figure 5. For it, our formula gives (see (8.22) in the document ProblsAlpha.pdf in https://github.com/biocom-uib/prob-alpha)

(21) $$\begin{array}{c} P_{\alpha ,8}\left(\;\tilde{T}\;\right)=\frac{{\left(1-\alpha \right)}^{2}\left(2-\alpha \right)}{126\left(7-\alpha \right)\left(6-\alpha \right)\left(5-\alpha \right)\left(3-\alpha \right)} \end{array}$$

while expression (19) assigns to $\tilde{T}$ a probability of half this value:

(22) $$\begin{array}{c} \frac{{\left(1-\alpha \right)}^{2}\left(2-\alpha \right)}{252\left(7-\alpha \right)\left(6-\alpha \right)\left(5-\alpha \right)\left(3-\alpha \right)}. \end{array}$$

This last value cannot be right, for several reasons. Firstly, by [4, §3.12], when *α* = 1/2, Ford's model is equivalent to the uniform model, where every cladogram in *𝒯*_*n*_ has the same probability

(23) $$\begin{array}{c} \frac{1}{\left|BT_{n}\right|}=\frac{1}{\left(2n-3\right)!!} \end{array}$$

and when *α* = 0, Ford's model gives rise to the Yule model [1, 14], where the probability of every *T* ∈ *𝒯*_*n*_ is

(24) $$\begin{array}{c} P_{Y}\left(\;T\;\right)=\frac{2^{n-1}}{n!}\underset{v\in V_{int}\left(T\right)}{\prod }\frac{1}{\kappa_{T}\left(v\right)-1}. \end{array}$$

In particular, $P_{1/2,8}(\tilde{T})$ should be equal to 1/135135 and $P_{0,8}(\tilde{T})$ should be equal to 1/19845. Both values are consistent with our formula, while expression (22) yields half these values.

As a second reason, which can be checked using a symbolic computation program, let us mention that if we take expression (22) as the probability of $\tilde{T}$ and hence of all other cladograms with its shape, and we assign to all other cladograms in *𝒯*_8_ the probabilities computed with Proposition 2, which agree on them with the values given by (19) (they are also provided in the aforementioned document ProblsAlpha.pdf), these probabilities do not add up 1.

Combining Proposition 2 and (4) we obtain the following result.

**Corollary 4 .**

For every *T* ^*∗*^ ∈ *𝒯*_*n*_^*∗*^ with *k* symmetric branch points,

(25) $$\begin{array}{c} P_{\alpha ,n}^{\left(*\right)}\left(\;T^{*}\;\right)=\frac{2^{n-k-1}}{\Gamma_{\alpha }\left(n\right)}\underset{\left(a,b\right)\in NS\left(T^{*}\right)}{\prod }\varphi_{\alpha }\left(\;a,b\;\right). \end{array}$$

This formula does not agree, either, with the one given in [4, Proposition 29]: the difference lies again in the same factor of 2 to the power of the number of internal nodes that are not symmetric branch points but whose children have the same number of descendant leaves.

The family of density mappings (*P*_*α*,*n*_)_*n*_ satisfies the following Markov branching recurrence.

**Corollary 5 .**

For every 0 < *m* < *n* and for every *T*_*m*_ ∈ *𝒯*_*m*_ and *T*_*n*−*m*_ ∈ *𝒯*_*n*−*m*_,

(26) Pα,nTm⋆Tn−m=2qαm,n−mnmPα,mTmPα,n−mTn−m.

**Proof**

If *T*_*m*_ ∈ *𝒯*_*m*_ and *T*_*n*−*m*_ ∈ *𝒯*_*n*−*m*_, then

(27) Pα,mTm=2m−1m!Γαm∏a,b∈NSTmφαa,bPα,n−mTn−m=2n−m−1n−m!Γαn−m·∏a,b∈NSTn−mφαa,b,Pα,nTm⋆Tn−m=2n−1n!Γαn∏a,b∈NSTm⋆Tn−mφαa,b=2n−1n!Γαnφαm,n−m∏a,b∈NSTmφαa,b·∏a,b∈NSTn−mφαa,b=2n−1n!Γαnφαm,n−m·m!Γαm2m−1Pα,mTm·n−m!Γαn−m2n−m−1·Pα,n−mTn−m=2qαm,n−mnmPα,mTm·Pα,n−mTn−m

as we claimed.

**Remark 6 .**

Against what is stated in [4], (*P*_*α*,*n*_^(*∗*)^)_*n*_ does not satisfy any Markov branching recurrence; that is, there does not exist any symmetric mapping *Q* : *ℤ* ^+^ × *ℤ* ^+^ → *ℝ* such that, for every *k*, *l*⩾1 and for every *T*_*k*_ ∈ *𝒯*_*k*_^*∗*^ and *T*_*l*_ ∈ *𝒯*_*l*_^*∗*^,

(28) $$\begin{array}{c} P_{\alpha ,k+l}^{\left(*\right)}\left(\;T_{k}⋆T_{l}\;\right)=Q\left(\;k,l\;\right)\cdot P_{\alpha ,k}^{\left(*\right)}\left(\;T_{k}\;\right)\cdot P_{\alpha ,l}^{\left(*\right)}\left(\;T_{l}\;\right). \end{array}$$

**Figure 6:**
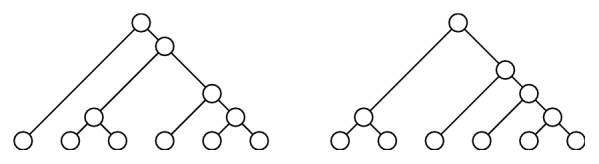
The tree shapes in *𝒯*_6_^*∗*^ mentioned in Remark 6.

Indeed, let $T_{m}^{*},{\hat{T}}_{m}^{*}\in {𝒯}_{m}^{*}$ be any two different tree shapes, both with *m* leaves and *k* symmetric branch points, for instance, the tree shapes in *𝒯*_6_^*∗*^ depicted in Figure 6. Then,

(29) Pα,m∗Tm∗=2m−k−1Γαm∏a,b∈NSTm∗φαa,b,Pα,m∗T^m∗=2m−k−1Γαm∏a,b∈NST^m∗φαa,b.

In this case, *T*_*m*_^*∗*^⋆*T*_*m*_^*∗*^ ∈ *𝒯*_2*m*_^*∗*^ has 2*k* + 1 symmetric branch points and therefore

(30) Pα,2m∗Tm∗⋆Tm∗=22m−2k−2Γα2m∏a,b∈NSTm∗⋆Tm∗φαa,b=22m−2k−2Γα2mφαm,m∏a,b∈NSTm∗φαa,b2=22m−2k−2Γα2mφαm,mΓαm2m−k−1Pα,m∗Tm∗2=qαm,mPα,m∗Tm∗Pα,m∗Tm∗

while $T_{m}^{*}⋆{\hat{T}}_{m}^{*}\in {𝒯}_{2m}^{*}$ has 2*k* symmetric branch points and therefore

(31) Pα,2m∗Tm∗⋆T^m∗=22m−2k−1Γα2m∏a,b∈NSTm∗⋆T^m∗φαa,b=22m−2k−1Γα2mφαm,m∏a,b∈NSTm∗φαa,b·∏a,b∈NST^m∗φαa,b=22m−2k−1Γα2mφαm,m·Γαm2m−k−1Pα,m∗Tm∗·Γαm2m−k−1Pα,m∗T^m∗=2qαm,mPα,m∗Tm∗Pα,m∗T^m∗

and *q*_*α*_(*m*, *m*) ≠ 2*q*_*α*_(*m*, *m*). This shows that there does not exist any well-defined, single real number *Q*(*m*, *m*) such that

(32) Pα,2m∗T1,m∗⋆T2,m∗=Qm,m·Pα,m∗T1,m∗·Pα,m∗T2,m∗

for every *T*_1,*m*_^*∗*^, *T*_2,*m*_^*∗*^ ∈ *𝒯*_*m*_^*∗*^.

## Data Availability

The data used to support the findings of this study are available at the Github page that accompanies this paper.

## References

[1] Yule G. U. (1925). A Mathematical Theory of Evolution, Based on the Conclusions of Dr. J. C. Willis, F.R.S.. *Philosophical Transactions of the Royal Society B: Biological Sciences*.

[2] Aldous D., Aldous D., Pemantle R. (1996). Probability distributions on cladograms. *Random discrete structures*.

[3] Blum M. G. B., François O. (2006). Which random processes describe the tree of life? A large-scale study of phylogenetic tree imbalance. *Systematic Biology*.

[4] Ford D. J. Probabilities on cladograms: Introduction to the alpha model. https://arxiv.org/abs/math/0511246.

[5] Keller-Schmidt S., Tuğrul M., Eguíluz V. M., Hernández-García E., Klemm K. (2015). Anomalous scaling in an age-dependent branching model. *Physical Review E: Statistical, Nonlinear, and Soft Matter Physics*.

[6] Kirkpatrick M., Slatkin M. (1993). Searching for Evolutionary Patterns in the Shape of a Phylogenetic Tree. *Evolution*.

[7] Popovic L., Rivas M. (2016). Topology and inference for Yule trees with multiple states. *Journal of Mathematical Biology*.

[8] Sainudiin R., Véber A. (2016). A Beta-splitting model for evolutionary trees. *Royal Society Open Science*.

[9] Mooers A. O., Heard S. B. (1997). Inferring evolutionary process from phylogenetic tree shape. *The Quarterly Review of Biology*.

[10] Felsenstein J. (2004). *Inferring Phylogenies*.

[11] Coronado T. M., Mir A., Rosselló F., Valiente G. A balance index for phylogenetic trees based on quartets. https://arxiv.org/abs/1801.05411.

[12] SageMath, the Sage Mathematics Software System (Version 7.6), The Sage Developers (2017) http://www.sagemath.org

[13] Cavalli-Sforza L. L., Edwards A. W. (1967). Phylogenetic Analysis: Models and Estimation Procedures. *Evolution*.

[14] Harding E. F. (1971). The probabilities of rooted tree-shapes generated by random bifurcation. *Advances in Applied Probability*.

